# EFL Teachers’ Mindfulness and Emotion Regulation in Language Context

**DOI:** 10.3389/fpsyg.2022.877108

**Published:** 2022-06-09

**Authors:** Na Wang

**Affiliations:** Jiao Zuo Teachers College, Jiaozuo, China

**Keywords:** EFL teachers, emotion regulation, educator, language context, mindfulness

## Abstract

The academic setting is a collection of diverse emotive needs that require skillful educators who can regulate and deal with them. Emotion regulation by language educators emphasizes the techniques that language educators use to regulate their emotions. In addition, English as a Foreign Language (EFL) teachers have a growing interest in examining the utilization of mindfulness-based methods to study and instruct a foreign language. Indeed, it is regarded as a strategy that has been connected to constructive encouragement when utilized as a managing technique for anxiety and worry. Concerning this, the goal of this mini review is to examine the function of mindfulness in regulating educators’ focus, emotion, manner, and contemplation. Some theoretical suggestions for educational situations are presented.

## Introduction

Educators constantly face a variety of happy and sad emotional experiences in the workplace and it is expected that during the severe flow of emotional experiences, they should be skilled enough in handling and controlling their emotional needs, since how educators interpret their emotions influences their decisions, teaching, and wellbeing (Heydarnejad et al., 2021; Pishghadam et al., 2021). As the center of learning, EFL teachers are broadly recognized to be exposed to a variety of feelings and inconveniences during their career, which is regarded as one of the most challenging professions (Benevene et al., 2020). Scholastic achievement and educator presentation depend heavily on the capability of recognizing and managing such feelings, as they must confront educational, cultural, emotional, and spiritual difficulties simultaneously. Therefore, it can be contended that educators’ emotional regulation is related to their ability to deal with and maintain emotive experiences in the class (Li, 2021). Based on this, it can be maintained that educators’ emotion regulation denotes their capability to succeed and endure classroom emotive practices (Wang and Ye, 2021). Concerning EFL educator educators, this competence to control feelings is an important socio-emotive ability that provides resilience and pliability in dealing with the unfavorable circumstances that often occur during instruction (Wijaya, 2021). Undoubtedly, an integral component of daily life is emotional regulation. It is characterized as endeavors made by people to affect what feelings they have when they have them, and how they are encountered and portrayed (Gross, 2015). This capability can be attained using a variety of techniques, based on evolving timing of the sensation; thus, regulation techniques can be reaction-oriented or proactive-oriented (Greenier et al., 2021). Moreover, EFL educators can up-regulate their feelings to enhance instructional effectiveness and manage teaching assignments. Similarly, they can down-regulate feelings to prevent adverse effects on students’ class engagement, contribution, presentation, or inspiration (Gong et al., 2013). In other words, emotional regulation influences the process by which educators change their emotions (Gross, 2015). Education encompasses both grasping subject matter messages to learners and controlling the emotive aspects of teaching (Richards, 2020). That is, educators’ high level of emotions regulation, leads to higher ability in using their mental capacities (Heydarnejad et al., 2021).

Mindfulness has been regarded as a supporter of emotion regulation process (Santos Alves Peixoto et al., 2021). Mindfulness is defined as the consciousness arising from deliberate attention in the current time, without judgment as to the unfolding of moment-to-moment experiences (Kabat-Zinn, 2004). Recent research indicates that mindfulness has a positive impact on human health, reduces distress intensity, accelerates emotional recovery, and improves the power to participate in purposeful behaviors (MacDonald and Baxter, 2017). The mindfulness construct has become central to researchers, due to the increased interest in positive outcomes related to mindfulness, like emotional, psychological, and social wellbeing and it also reduce symptoms of psychological distress (Bowlin and Baer, 2012). In training mindfulness, one will know how to self-regulate his or her attention and try to observe mental and emotional patterns while remaining open and receptive without overlooking upsetting experiences (Iani et al., 2019). Academic researchers have focused on mindfulness, and recent research has shown that mindfulness can affect behavior (Charoensukmongkol, 2016).

Recently, as stated by Pennock and Alberts (2016), strong evidence has been presented by psychology indicating that mindfulness and issues such as “positive affect,” “satisfaction with life” and general “wellbeing” are interconnected. Positive psychology (PP) is a scientific study of constructive attributes that support the prosperity of people and societies (Zhang and Zhang, 2020; Wang et al., 2021). The concept of mindfulness refers to the capability of paying attention to experiences with objective curiosity and admittance, generally, curiosity about mindfulness is becoming popular and the term, known as PP intervention, is being applied in many fields, and education is the most recent example (Kabat-Zinn, 2004).

As interest in language educators’ emotions increases, studies on educators’ emotional regulation have increased over recent years (Keller et al., 2014) and although emotion regulation is prominent in various research fields and specifically in the language teaching field, their focus is not on language educators (Talbot and Mercer, 2018; Fathi and Derakhshan, 2019; Bielak and Mystkowska-Wiertelak, 2020). Likewise, based on a review of the mindfulness research, it was revealed that mindfulness constantly led to remarkable results for its positive outcomes. For example, mindfulness may mitigate hopelessness and anxiety, stress, emotion regulation, and overall wellbeing and it also improves academic performance (Erbe and Lohrmann, 2015). However, the main intention and significance of this line of review is to study the above-mentioned constructs that necessitate further research in language education among educators that can be taken into consideration by language stakeholders due to the importance of PP.

## Review of Major Concepts

### Emotion Regulation

Emotions are core aspects of peoples’ personal and interpersonal lives, and experiences generated by emotions can both positively and negatively affect performance. Perhaps due to such strong influence, these experiences do not float freely, thus if you are to comprehend the pressures imposed on you from other people and societies, you need to be aware of them and investigate them (Nezlek and Kuppens, 2008; Wang and Guan, 2020). The term emotion regulation alludes to the capability of adjusting, managing, changing, and controlling the conception and portrayal of feelings as a result of inner and outer elements (Wijaya, 2021). It is the cycle through which individuals seek to influence the experience of emotive encounters to achieve their individual goals (Jennings et al., 2011). Emotion regulation refers to the procedures through which people affect their emotions, times of having them, and the way they experience and state their emotions (Huang et al., 2016). It is considered a socio-emotive concept aimed at sustaining and strengthening feelings and suppressing and adjusting them (Akbari et al., 2017). From another point of view, emotional regulation can be regarded as a personal difference attribute that is reasonably well-balanced and not time- or situation-dependent (Gross, 2015). The model of emotion regulation differentiates between reappraisal and suppression and Individual timing of intervention is the difference of both typical kinds of emotion regulation tactics, reevaluation, and suppression. Reevaluation takes place before emotions are stimulated by external stimuli, however, suppression occurs after the specific emotions are formed (Gross, 2015).

### Mindfulness

Prominent in the field of positive psychology, mindfulness has important advantages like enhancing working memory, advancing health, decreasing anxiety, etc. (Brown et al., 2007). The concept “mindfulness” means to be aware, pay attention and remember, the definition of which can be “continuous awareness,” which is physical freedom or continuous awareness experienced with no judgment (Davis and Hayes, 2011). Also, Mindfulness is characterized as focusing on and recognizing inner and outer encounters as they take place (Brown et al., 2007). Meditation and mindfulness-based coaching can elevate the degree of mindfulness. Based on Buddhist rituals, the exercise of mindfulness proposes that emotive distress can be relieved by being conscious of the current moment and building internal consciousness. Mindfulness has been demonstrated to constructively impact psychological and mental health (Baer et al., 2012). Mindfulness is conceptualized as an attribute and a condition that can be built through practice (Brown et al., 2007) and it is connected to a sense of empowerment and self-esteem and individuals with a great degree of mindfulness can normally be expected to better adjust their thoughts and emotions and enhance their self-effectiveness (Iani et al., 2019). Mindfulness includes two main mechanisms: self-adjustment of focus and unbiased consciousness of experience (Bishop et al., 2004). Regulation of emotion encourages consciousness of the emotive, intellectual, and physical encounters that take place from time to time. Unbiased consciousness, described by interest, open-mindedness, and embracement of that knowledge can strengthen management by reducing responsiveness (Bishop et al., 2004).

## Conclusion

Constructed on the conclusions taken from the review of literature, some recommendations for school officials, experts, and scholars are presented. On the whole, the significance of developing mindfulness in processes of learning and teaching is highlighted by this mini-review. Mindfulness has to be done in an educational context because an effective educational system should ensure developing students’ self-fulfillment when learning and provide them with more achievement. Teaching mindfulness for educators also helps learners’ success (Jennings et al., 2011). Mindfulness is linked to numerous predictors of health that can be an important factor of interest. More mindful educators may be able to better self-regulate by providing a cushioning impact against inconveniences of controlling a class. Moreover, mindfulness-based mediations diminish nervousness and stress through nurturing consciousness in the current moment and concentrating on satisfactory intrinsic and extrinsic motivations.

## Implications

Mindfulness training for educators increases their wellbeing and also raises learners’ mindfulness; it also basically alters the relationships and the classroom environment. Similarly, it can be very helpful for EFL educators as mindfulness motivates them, make them more target-oriented to be successful in their jobs. In the same vein, mindfulness generally lessens anxiety and depression and helps lessen or even diminish language-related stress that has been consequently proved to lead to enhancing EFL presentation. To prepare the route for inducing highly useful self-help abilities, pre-service and in-service programs for training educators must be built, particularly in the language learning context. EFL syllabus designers are greatly suggested to integrate content materials that stimulate mindfulness followed by emotion regulation strategies.

Insufficient emotion regulation not only causes anxiety and depression in educators but also negatively affects learners due to the poor quality of teaching (McLean and Connor, 2015). The present review determined that the emotional regulation of language educators in all scholastic settings is crucial to the cycle of successful instructing and scholastic psychology. Teaching in school is one of the careers that particularly need emotion regulation skills needed to successfully manage difficult learner behaviors and overcome their emotional states (Skinner and Beers, 2016). Indeed, this review can be helpful for EFL educators, because it can raise awareness as well as the use of the right emotion regulation tactics to increase or decrease certain emotions in the classroom to promote better learning. Realizing the prominence of emotions in language learning, one can think extremely about their intrinsic moods and their students and develop proper methods of flourishing them. Also, teaching mindfulness may be employed as intervention which may support learners to have better commitment within the class and achieve better in English learning setting.

Because of the significance of emotion regulation in an academic setting, several studies are recommended on the role of such paradigm and other variables about PP. Since it is argued that teachers from different cultures employ perceptible and diverse emotion regulation approaches, cross-cultural and multicultural studies are also recommended to be conducted to consider this issue. Further studies are suggested to do longitudinal ones on EFL educators’ constructive emotion regulation with qualitative instruments to represent the progressive paths of regulatory strategies. Since the purpose of teachers and school officials concentrates on increasing standards of educators’ excellence, these concepts, namely mindfulness, and emotion regulation should be taken into account.

## Author Contributions

The author confirms being the sole contributor of this work and has approved it for publication.

## Conflict of Interest

The author declares that the research was conducted in the absence of any commercial or financial relationships that could be construed as a potential conflict of interest.

## Publisher’s Note

All claims expressed in this article are solely those of the authors and do not necessarily represent those of their affiliated organizations, or those of the publisher, the editors and the reviewers. Any product that may be evaluated in this article, or claim that may be made by its manufacturer, is not guaranteed or endorsed by the publisher.
